# Modeling Notch-Induced Tumor Cell Survival in the *Drosophila* Ovary Identifies Cellular and Transcriptional Response to Nuclear NICD Accumulation

**DOI:** 10.3390/cells10092222

**Published:** 2021-08-27

**Authors:** Allison Jevitt, Yi-Chun Huang, Su-Mei Zhang, Deeptiman Chatterjee, Xian-Feng Wang, Geng-Qiang Xie, Wu-Min Deng

**Affiliations:** 1 Department of Biological Science, Florida State University, Tallahasee, FL 32306, USA; jevitta@omrf.org (A.J.); zhangsumei@ahmu.edu.cn (S.-M.Z.); gengqiang.xie@med.fsu.edu (G.-Q.X.); 2 Department of Biochemistry and Molecular Biology, Tulane University School of Medicine, New Orleans, LA 70112, USA; yhuang30@tulane.edu (Y.-C.H.); dchatterjee@tulane.edu (D.C.); xwang52@tulane.edu (X.-F.W.)

**Keywords:** *Drosophila*, Notch, NICD, follicle, epithelium, nuclear, accumulation, tumorigenesis, apoptosis, survival

## Abstract

Notch is a conserved developmental signaling pathway that is dysregulated in many cancer types, most often through constitutive activation. Tumor cells with nuclear accumulation of the active Notch receptor, NICD, generally exhibit enhanced survival while patients experience poorer outcomes. To understand the impact of NICD accumulation during tumorigenesis, we developed a tumor model using the *Drosophila* ovarian follicular epithelium. Using this system we demonstrated that NICD accumulation contributed to larger tumor growth, reduced apoptosis, increased nuclear size, and fewer incidents of DNA damage without altering ploidy. Using bulk RNA sequencing we identified key genes involved in both a pre- and post- tumor response to NICD accumulation. Among these are genes involved in regulating double-strand break repair, chromosome organization, metabolism, like *raptor*, which we experimentally validated contributes to early Notch-induced tumor growth. Finally, using single-cell RNA sequencing we identified follicle cell-specific targets in NICD-overexpressing cells which contribute to DNA repair and negative regulation of apoptosis. This valuable tumor model for nuclear NICD accumulation in adult *Drosophila* follicle cells has allowed us to better understand the specific contribution of nuclear NICD accumulation to cell survival in tumorigenesis and tumor progression.

## 1. Introduction

The Notch pathway is a conserved developmental signaling pathway that coordinates essential cellular processes in metazoans such as differentiation, pattern formation, cell-cycle progression, morphogenesis, migration, and apoptosis. Canonical Notch signaling occurs at the cellular membrane of a signal-receiving cell upon direct contact with a signal-sending cell through the binding of Delta/Serrate/LAG-2 (DSL) ligand to the trans-membrane Notch (N) receptor [1]. Following activation, Notch undergoes two proteolysis events. First, ADAM protease cleaves the extracellular domain (NECD), forming the intermediary, membrane-tethered Notch Extracellular Truncation (NEXT) [2]. In a second cleavage by γ-secretase, the active receptor Notch Intracellular Domain (NICD) is released. NICD is transported to the nucleus through the endocytosis pathway where it creates a complex with the DNA binding protein, Suppressor of Hairless (Su(H)), and the coactivator, Mastermind (Mam) and regulates downstream targets [3,4]. NICD, like other short-lived proteins, has a rich in proline (P), glutamic acid (E), serine (S) and threonine (T) residues (PEST) domain which controls its rapid turnover through ubiquitin-mediated proteolysis [5]. The short half-life of NICD makes the Notch pathway highly dose-dependent. Activation and degradation of the Notch receptor (NICD) must be carefully regulated to ensure proper cellular function across different cellular and developmental contexts.

In many types of cancers, mutations of the Notch negative regulatory region (NRR) and PEST domain result in the accumulation of NICD in the nucleus [6,7]. Nuclear retention of NICD is associated with increased tumor cell growth and cell survival, especially within the context of chemotherapy treatments [6,7,8,9]. Given the multifactorial nature of tumorigenesis and the context-dependent nature of Notch signaling, it is challenging to identify the cellular mechanisms by which NICD accumulation promotes cell survival and tumor progression. To tackle this, we utilized the genetic tools available in *Drosophila* to model NICD accumulation in tumors generated in the adult ovarian follicular epithelium. To validate this system, we demonstrated that ectopic NICD accumulates in tumor cell nuclei and protects them from apoptosis. With this model system established, we used it to identify the cellular phenotypes associated with nuclear accumulation of NICD. Notably, tumor cells with ectopic NICD expression had greater nuclear size heterogeneity and fewer marks of DNA damage than tumor cells without ectopic NICD expression. We also used bulk RNA sequencing to identify the large-scale transcriptional impacts of nuclear NICD accumulation pre- and post-tumor formation. Finally, single-cell RNA sequencing was used to identify the cell-type-specific gene expression patterns in NICD-overexpressing tumor cells. We found that in the mitotic follicle cells that form the tumor mass, NICD upregulates genes which promote DNA damage repair genes and suppress apoptosis.

## 2. Materials and Methods

### 2.1. *Drosophila* Strains and Culture Conditions

For all experiments, we used a Gal4 under the control of traffic jam (tj), in a somatic cell-specific pattern (tj-Gal4) with a temperature-sensitive control (Gal80^TS^). The full genotypes for the four main samples used in this study described here in detail. Control = tj-Gal4, Gal80^TS^, UAS GFP/CyO; Dr/ TM6B. NICD OE = tj-Gal4, Gal80^TS^, UAS GFP/ UAS NICD; Dr/ TM6B. L(2)gl KD = tj-Gal4, Gal80^TS^, UAS GFP/ CyO; UAS l(2)gl^IR^, UAS Dcr2/ TM6B. NICD OE + l(2)gl KD = tj-Gal4, Gal80^TS^, UAS GFP/ UAS NICD; UAS l(2)gl^IR^, UAS Dcr2/ TM6B. Flies were raised and crossed on standard medium with access to yeast at 18 ${}^{\circ }$C until time for tumor induction using the TARGET system [10]. After three days post pupal eclosion, progeny from crosses were moved to 29 ${}^{\circ }$C for three days to drive transgene expression unless stated otherwise.

Fly lines from Bloomington *Drosophila* Stock Center (BDSC), Vienna Drosophila Resource Center (VDRC), and Kyoto Stock Center (DGRC) used in this study are as followed: w^1118^ (BDSC:3605), tj-Gal4 (DGRC:104055), tub-Gal80^TS^ (BDSC:7017), UAS-l(2)gl-RNAi (VDRC: 51247), UAS-GFP (BDSC:4775), UAS-raptor-RNAi (BDSC:41912), and UAS-Dcr2 (BDSC:24651). The UAS-NICD line used in this study was kindly provided by the Schüpbach lab [11].

### 2.2. Immunofluorescence and Imaging

Ovaries were dissected in PBS, fixed for 15 min in 4% formaldehyde, washed 3 times in PBT, and stained with DAPI (Invitrogen). Anti-NICD (DSHB, mouse, 1:15), anti-γ-H2AV (DSHB, mouse, 1:200), anti-DCP1 (Cell Signaling, mouse, 1:200), and anti-PH3 (Millipore, rabbit, 1:200) primary antibodies were used. Alexa Fluor 546-conjugated goat anti-rabbit and anti-mouse, and 633-conjugated anti-mouse secondary antibodies were all used at 1:400 (Invitrogen). Samples were then mounted on a slide in a 80% glycerol mounting solution. All images were captured using the Zeiss LSM 800 confocal microscope and associated Zeiss microscope software (ZEN blue).

### 2.3. Segmentation and 3D Volume Measurements

To measure 3D volume of the follicular epithelium, confocal images of follicle-cell specific GFP expression (tj^TS^ > GFP) were analyzed using the Segmentation Editor in Fiji Is Just ImageJ (FIJI) [12]. Confocal z-stacks were first imported into FIJI using the Bio-Formats Import Option with separated channels. Tumors, marked by GFP, were manually selected using the brush tool. Rough selection of every few z-slices was done first, followed by interpolation in the segmentation editor, and automatic threshold selection. The volume measurements of the 3D selections were analyzed using MorphoLibJ [13].

### 2.4. Nuclear Area Measurements

Nuclear area was measured using the “draw spline contour” tool in the ZEN Blue Zeiss microscope software. Nuclear measurements were taken using DAPI staining at the z-slice with the widest nuclear area for each cell.

### 2.5. Allograft Methods

Previously established methods were used to allograft ovariole tissue into young, w^1118^ host flies three days after eclosion [14]. During the three days leading up to allograft implantation, females had access to male flies and yeast. To prepare allograft tissues, female flies of the desired genotype were sorted three days after pupal eclosion and incubated in 29 ${}^{\circ }$C for five days to permit transgene expression and tumor growth. Ovaries were dissected in complete medium (Grace’s Insect Basal Medium supplemented with 15% FBS). Using forceps, the posterior of each ovary was carefully removed and single ovarioles were gently teased apart. Allograft tissue consisting of a single ovariole (from germarium to stage 9) was then implanted into the abdomen of each host fly. Following allograft implantation, host flies were left to recover for 24 h at room temperature and then incubated in 29 ${}^{\circ }$C for 15 days with access to a (w^1118^) male and yeast. After 15 days, dissected abdominal tissue was fixed, stained, and imaged according to Immunofluorescence and Imaging methods above.

### 2.6. Flow Cytometry Analysis of DNA Content

Ovaries were dissected and dissociated into a single-cell suspension as previously described [15]. Single cells were fixed in a mixture of 500 μL 4% formaldehyde and 1 μL Vybrant DyeCycle Violet stain (Invitrogen) for 30 min at room temperature. Cells were pelletized and washed in 500 μL EBSS and analyzed using a FACSCanto cell analyzer. An excitation at 407 nm for Vybrant DyeCycle stain and at 488 nm for GFP was used.

### 2.7. RNA Sequencing

All flies for sequencing were reared in 18 ${}^{\circ }$C and then transferred to 29 ${}^{\circ }$C for either 24 or 96 h to generate pre- and post-tumor conditions, respectively. All flies had access to males and were given yeast supplement for 24 h prior to dissection.

Tissues were dissected from 40 flies in complete medium (Grace’s Insect Basal Medium supplemented with 15% FBS). The anterior section of the ovary was removed. Samples were transferred to a sterile Eppendorf tube and the media was aspirated before freezing samples in liquid nitrogen. Samples were stored until the day of library preparation in −80 ${}^{\circ }$C.

Total RNA was extracted using Trizol and libraries were made using the NEBNext Ultra II Directional RNA library Prep Kit for Illumina using the established protocol for use with NEBNext Poly(A) mRNA Magnetic Isolation Module (NEB#E7490). We used Rapid Run OBCG single read 50 bp on the Illumina HiSeq 2500 system to sequence these libraries with 2 biological replicates and 2 technical replicates for each time point and genotype. Reads were demultiplexed and indexes removed using CASAVA v1.8.2 (Illumina, USA).

### 2.8. RNA Sequencing Analysis

RNA reads of adequate quality were aligned to the *Drosophila melanogaster* Release 6 reference genome assembly [16]. FeatureCounts and DESeq2 were then used to assign gene names to genomic features and analyze fold change (log2(FC)) from count data [17,18]. GO enrichment analysis was performed using g:Profiler with a threshold of 0.05 [19].

### 2.9. Single-Cell RNA Sequencing and Analysis

Ovaries were collected from 50 adult flies, dissociated and filtered to isolate single cells according to established protocols [15]. Single-cell libraries for all samples were then generated using the Single Cell Bead Kit Cell 3${}^{\prime }$ Library & Gel Bead Kit v2 according to the protocol supplied by 10X Genomics. Raw sequencing reads from all libraries were processed using Cell Ranger (version 3.0.0) as recommended by Chromium single-cell gene expression software suite. The *Drosophila melanogaster* Release 6 reference genome was used from the Ensembl genome database [16]. Filtering, barcoding counting, and UMI counting was then performed using the multidimensional matrix.

Downstream analysis was performed on the Cell Ranger output using Seurat (Version 4.0) [20]. The raw data for each genotype were processed according to standard pre-processing workflow including selection of clusters with min.cell = 3 and min. features = 200. We then performed the SCTransform function for each dataset separately, which normalizes, scales and finds variable features. The standard Seurat workflow was then followed to identify integration features and perform data integration between all four datasets. A Principal Component Analysis (PCA) was then performed using dimensions = 1:10, and plotted the combined data using a Uniform Manifold Approximation and Projection (UMAP) using 1:10 dimensions. During the integration, shared expression across all samples (integration features) were used to group similar clusters across all genotypes. The established biological markers were then used to identify clusters in the control dataset [15].

## 3. Results

### 3.1. Modeling Nuclear Nicd Accumulation in Tumor Cells

**Figure 1:**
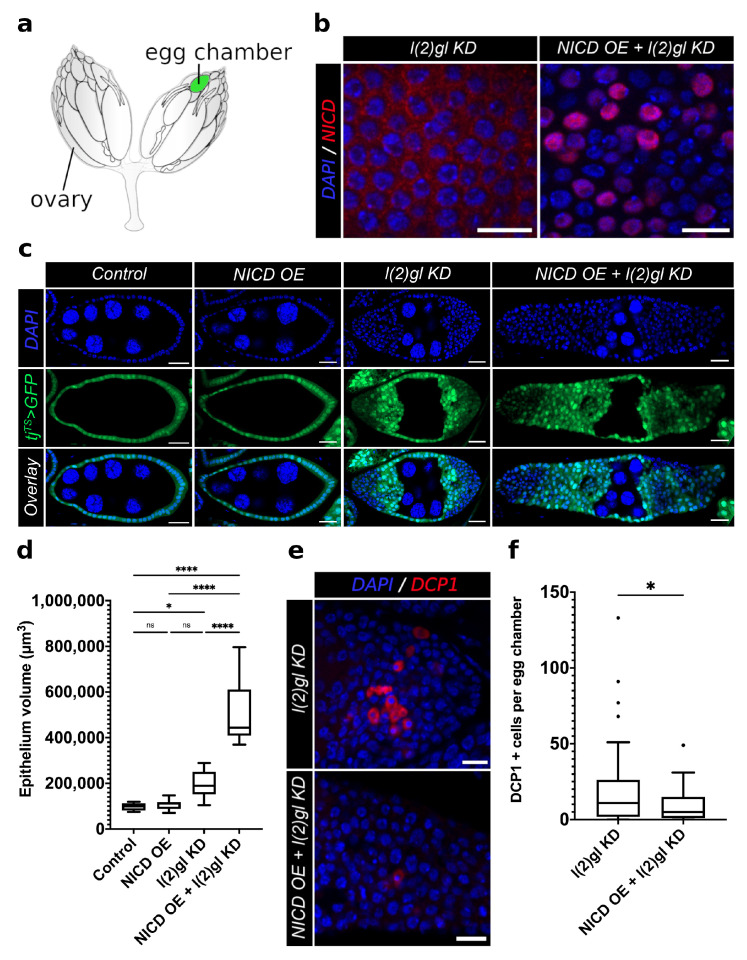
Nuclear accumulation of ectopic NICD promotes tumor growth and tumor cell survival in follicle cells. (**a**) An illustration of adult *Drosophila* ovaries connected by the oviduct. The position of a post-mitotic, mid-staged egg chamber is marked in green. (**b**) Confocal images comparing NICD antibody staining (red) patterns between l(2)gl KD and NICD OE + l(2)gl KD tumor cells. Both images are of post-mitotic, stage-7 egg chambers following three days of Gal4 activation in 29 ${}^{\circ }$C. NICD accumulates in high levels in NICD OE + l(2)gl KD cell nuclei compared to l(2)gl KD. (**c**) Confocal images of stage-7 egg chambers from each genetic background after 3 days of Gal4 activation in 29 ${}^{\circ }$C. The tissue-specific expression of tj^TS^ > tj-Gal4, Gal80^TS^, UAS GFP (GFP) marks all follicle cells in green, and DAPI marks all cell nuclei in blue. Scale bar = 20 μm. (**d**) Box and whisker plot comparing epithelial volume between genotypes. N = 10 egg chambers from different individuals. Comparisons from the post hoc Tukey HSD are shown. (Ns) not significant, (*) *p* < 0.05, (****) *p* < 0.0001. (**e**) Confocal images comparing apoptosis marker, DCP1 (red), between l(2)gl KD and NICD OE + l(2)gl KD genotypes. Scale bar = 20 μm. (**f**) Box and whisker plot of total number of DCP1-positive apoptotic cells per egg chamber. N = 50 egg chambers from 14 individuals. Significance value from *t*-test is shown (* = *p* < 0.05).

*Drosophila melanogaster*, has provided many valuable tumor models contributing to our understanding of cancer biology [21]. Here we used the follicle cells as an epithelial model system to study tumorigenesis. During oogenesis, follicle cells surround the germline cells in a monolayer forming a single developmental unit called an egg chamber (Figure 1a). As egg chambers exit the germarium, where stem cells are housed, they develop in a queue called an ovariole. Each ovary is composed of 16–18 ovarioles which each support the development of one egg at a time [22]. The entire process takes approximately a week leading up to ovulation [23]. Once a mature egg is released into the oviduct, the follicle cell layer remains in the ovary as a corpus luteum [23,24].

**Figure A1:**
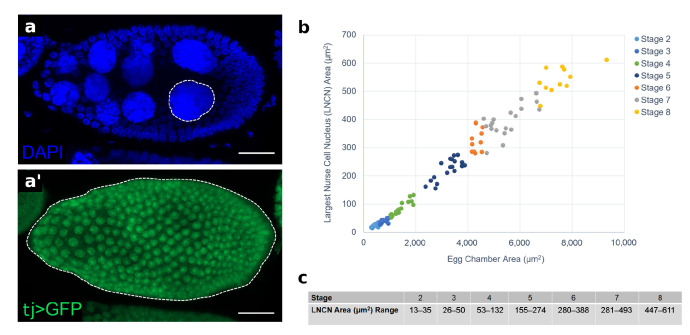
Egg chamber stage identification using the largest nurse cell nuclear area. (**a**) Confocal projection of a stage 8 egg chamber centered on the largest nurse cell nucleus (LNCN) marked with blue (DAPI). The dotted white line illustrates how area measurements were taken at the widest point of the LNCN of a projection. (**a’**) Confocal projection of te entire egg chamber with follicle-cell-specific expression of GFP driven by tj-Gal4 (green). Dotted line illustrates how measurements were taken of egg chamber area at the widest point of the projection. (**b**) Standard curve of egg chambers from stages 2–8. There is a positive, linear relationship between egg chamber area and LNCN area. For stages 2–5 and 7, n = 20. For stages 6 and 8, n = 11. (**c**) Table of LNCN area ranges for each stage. Scale bar = 20 μm.

We used the temporal and regional gene expression targeting (TARGET) system with a follicle-cell-specific Gal4 driver under the regulation of traffic jam (tj). Using temperature shifts, Gal4 expression was permitted for 3–5 days to allow for enough time for egg chambers to form and grow to post-mitotic stages. Post-mitotic (stage 6–8) follicle cells were then observed for phenotypic and transcriptional changes. We used the size of germline nuclei to determine the stage of egg chambers since they remain unaffected by the tj-Gal4 (Figure A1). Previous work has shown that loss of the neoplastic tumor suppressor, *lethal (2) giant larvae (l(2)gl*), in the follicle cells induces tumor growth at both the anterior and posterior termini of egg chambers [25]. The tumor suppressor activity of l(2)gl is functionally conserved to the human homolog called Hugl-1. Hugl-1 levels are depleted in human cancer originating from the colon, breast, prostate, lung, skin, and ovary [26,27,28].

**Figure A2:**
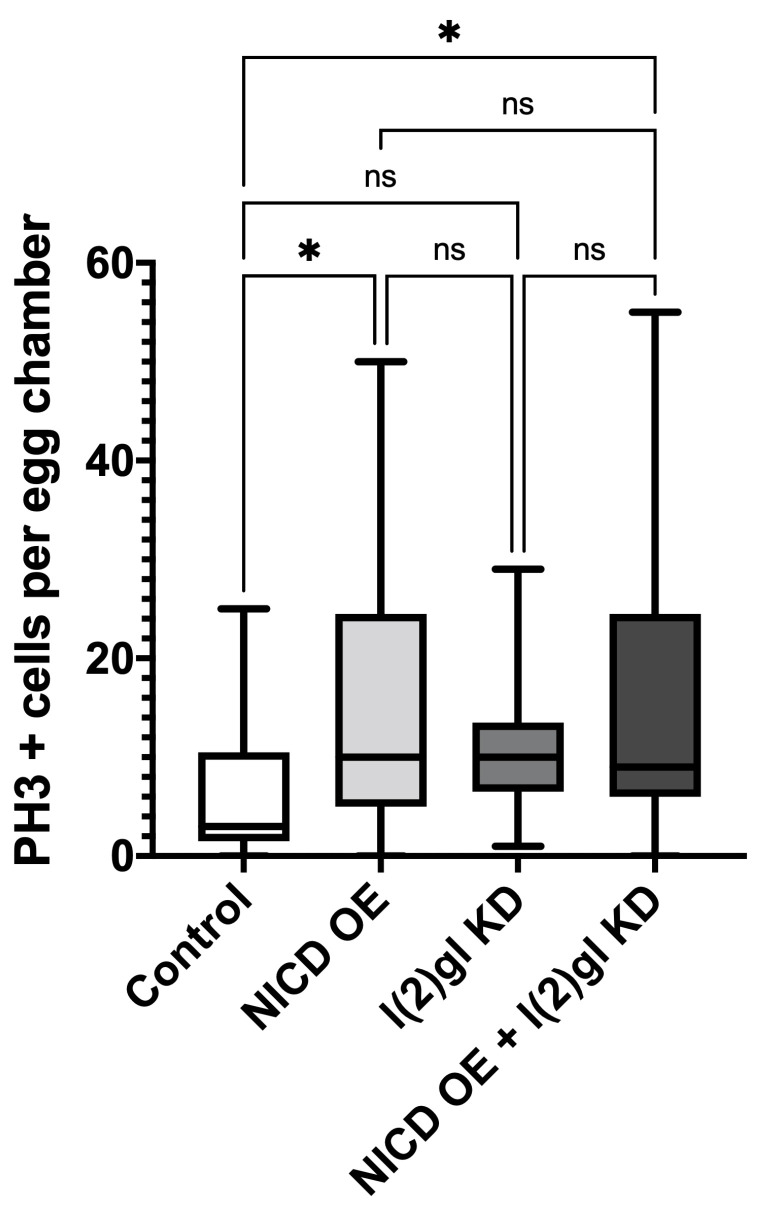
Comparison of proliferation between NICD and non-NICD-overexpressing gentoypes. Box and whisker plot comparing number of PH3 + cells were counted per egg chamber. Comparisons from the post hoc Tukey HSD are shown. (ns) not significant, (*) *p* < 0.05. N = 20 egg chambers from at least 5 individuals per condition. Genotypes with NICD OE have more proliferation than those without.

**Figure A3:**
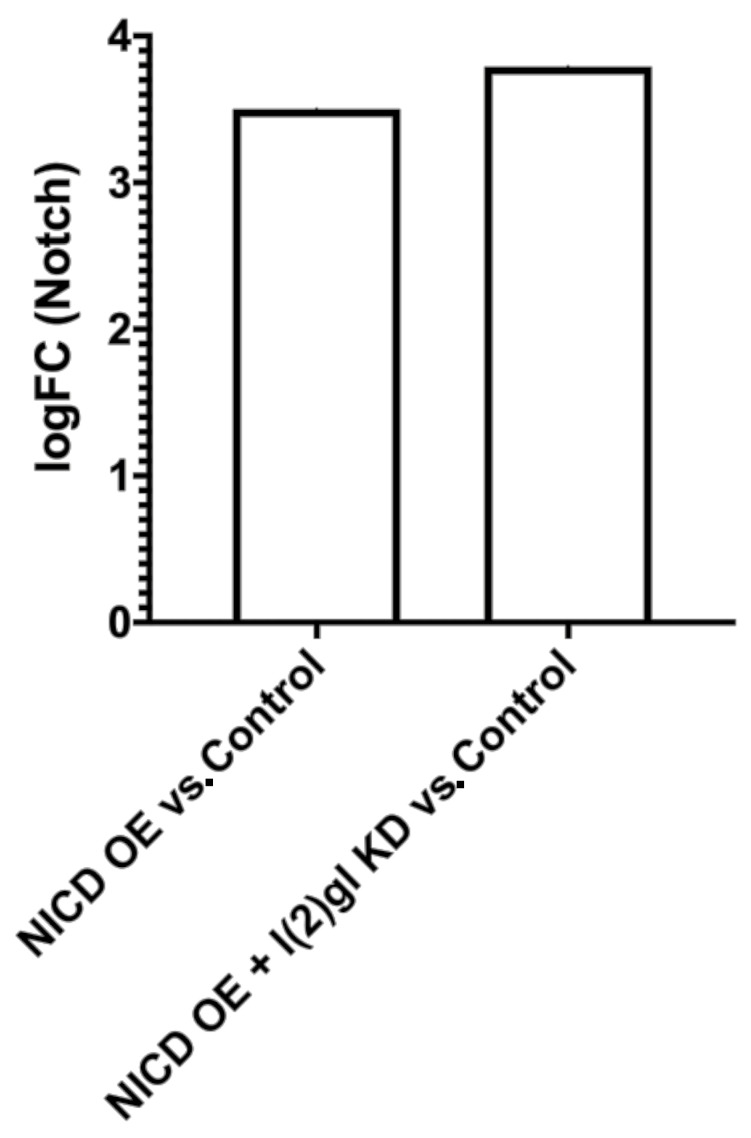
Comparable levels of Notch expression (dosage) between NICD OE and NICD OE + l(2)gl KD genotypes compared to Control. LogFC Notch expression between NICD OE and NICD OE + l(2)gl KD compared to Control from bulk RNA sequencing experiment.

To understand the role of NICD accumulation during tumorigenesis we knocked down *l(2)gl* (*l(2)gl* KD) while simultaneously overexpressing NICD (NICD OE) using the temperature-sensitive, follicle-cell-specific tj-Gal4 driver, marked by GFP expression (tj^TS^ > GFP). As shown in Figure 1b, under these conditions, NICD indeed accumulated at high levels in tumor cell nuclei. We observed that while NICD is expressed evenly across the follicular epithelium, as marked by UAS GFP, there is heterogeneity in the levels of NICD accumulation (Figure 1b,c). While NICD overexpression alone did not generate follicle cell tumors, ectopic NICD expression in l(2)gl KD tumor cells (hereby referred to NICD, l(2)gl +/− cells), promoted increased tumor growth, resulting in significantly larger tumors (Figure 1c,d). Quantification of proliferation revealed that NICD-overexpressing tissues had increased numbers of dividing cells, marked by PH3, compared to controls (Figure A2). To ensure that this model system recapitulated the cell survival phenotypes reported in human cancers, we compared the numbers of apoptotic cells in NICD, l(2)gl +/− and l(2)gl +/− tumors [29]. Since Notch is known to be dose dependent, we also confirmed that the level of NICD overexpression was comparable between our NICD and NICD, l(2)gl +/− conditions despite differing numbers of UAS transgenes. Using RNA sequencing after 24 hours of NICD overexpression, we found that Notch was similarly upregulated in NICD (logFC = 3.50) and NICD, l(2)gl +/− (logFC = 3.79) conditions compared to controls (Figure A3).

**Figure 2:**
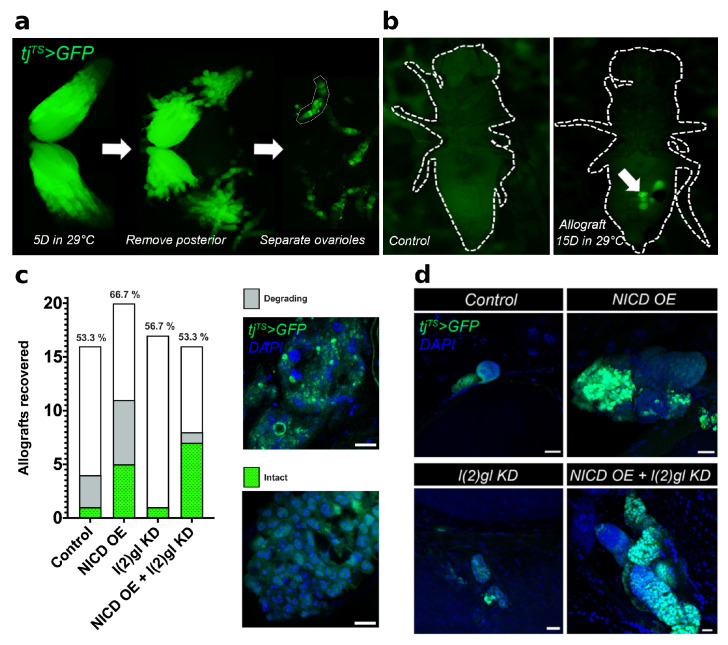
Cells with ectopic NICD expression gain a long-term survival advantage. (**a**) Fluorescent image from a dissecting microscope showing the steps for allograft preparation. First, Gal4 activity was initiated by transferring three-day-old female flies to permissive temperature (29 ${}^{\circ }$C) for five days. Ovaries with follicle cell tj^TS^ > GFP marker (green) were dissected (left), cut to remove the bulky posterior half of the ovaries including and following stage 10A (middle), and ovarioles were gently teased apart (right) for implantation into the host fly. (**b**) Fluorescent image of a host flies following implantation of NICD OE + l(2)gl KD tumor tissue (right) next to an uninjected control (left) following 15 days in 29 ${}^{\circ }$C. GFP-expressing (tj^TS^ > GFP) allograft tissue (green) was visible through the abdomen of the host fly (arrow). (**c**) Bar graph showing numbers of surviving flies. Within each bar, the number of flies which had GFP + allograft tissue is shown in green and gray. Green bars represent allograft tissue that was intact, characterized as having entire nuclei and cytoplasmic GFP expression. Gray bars represent degrading allograft tissue characterized by bright, punctate GFP and DAPI signal. (**d**) Confocal images of an intact allograft tissue from each genotype after 15 days in 29 ${}^{\circ }$C. DAPI marks nuclei. Scale bar = 20 μm.

To assess the long-term survival potential of NICD, l(2)gl +/− tumor cells compared to l(2)gl +/− cells, allograft tissues were implanted into the abdomen of wild-type female flies and observed for 15 days (Figure 2a,b). This 15 day period is nearly twice the time it takes to complete oogenesis, which typically lasts up to a week [22,30]. Surviving flies at the end of 15 days were dissected and the GFP-expressing allograft tissues were recovered, fixed, stained, and imaged to assay the long-term survival potential of cells overexpressing NICD (Figure 2a,b). Recovered allograft tissue was scored as either degrading, with bright punctate GFP and DAPI staining or intact, with clearly defined nuclei and cytoplasmic GFP (Figure 2c,d). Consistent with our expectations, we found that NICD, l(2)gl +/− allografts had a larger proportion of intact tissue with overall larger masses of cells compared to controls. Surprisingly, NICD OE cells had a similar survival ability to NICD, l(2)gl +/− tumor cells further indicating the role of NICD accumulation in promoting survival. (Figure 2c,d).

### 3.2. Ectopic Nicd Expression Promotes Increased Nuclear Size and Heterogeneity without Impacting Ploidy

**Figure 3:**
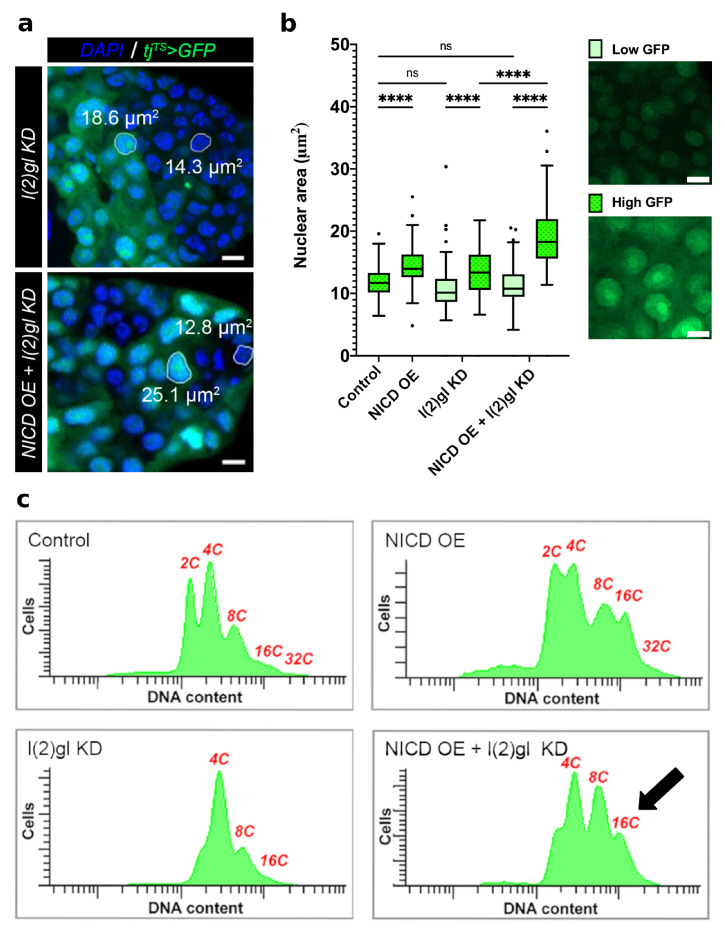
NICD accumulation promotes nuclear size heterogeneity. (**a**) Confocal image comparing nuclear size of tumor cells between l(2)gl KD and NICD OE + l(2)gl KD genotypes. Each image is of the posterior tumor of a mid-staged egg chamber. Example area measurements are shown for larger (high GFP-expressing) and smaller (low GFP-expressing) cell nuclei. Scale bar = 5 μm. (**b**) Box and whisker plot of nuclear area measurements between high and low GFP-expressing cells between Control, NICD OE, l(2)gl KD, and NICD OE + l(2)gl KD genotypes (left). Example images from NICD OE + l(2)gl KD show cells from the low GFP and high GFP groups without DAPI signal for a better comparison (right). Scale bar = 5 μm. N = 100 nuclei in 5 egg chambers from different individuals. Comparisons from the post hoc Tukey HSD are shown. (Ns) not significant, (****) *p* < 0.0001. (**c**) Flow cytometry analysis of DNA content for all genotypes. Copies of the genome are labeled (2-32C (GFP) in red. NICD expression increases the proportion of polyploid cells (8-16C), arrow.

During oogenesis, Notch activation at stage 6 promotes the mitotic to endocycle transition of follicle cells [23]. Following this transition, follicle cells undergo three rounds of endoreduplication, resulting in a ploidy of 16C [31]. We observed that NICD, l(2)gl +/− tumors had an increased nuclear size heterogeneity when compared to l(2)gl +/− tumors and that cells with higher GFP expression had larger nuclei and conversely cells with lower GFP expression had smaller nuclei (Figure 3a,b). Quantification of nuclear area in high and low GFP-expressing cells revealed that NICD, l(2)gl +/− tumor cells were much larger on average than l(2)gl +/− (Figure 3b). To understand how NICD, l(2)gl +/− tumor cell nuclei were increasing in size so dramatically: either due to additional rounds of endoreduplication or changes to nuclear organization and compaction, we analyzed DNA content of dissociated follicle cells using flow cytometry. We found that while follicle cells expressing ectopic NICD had an increased proportion of 8C and 16C cells, they did not exceed the expected copy number (Figure 3c).

### 3.3. Nicd-Overexpressing Tumor Cells Lack Marks of DNA Damage

**Figure 4:**
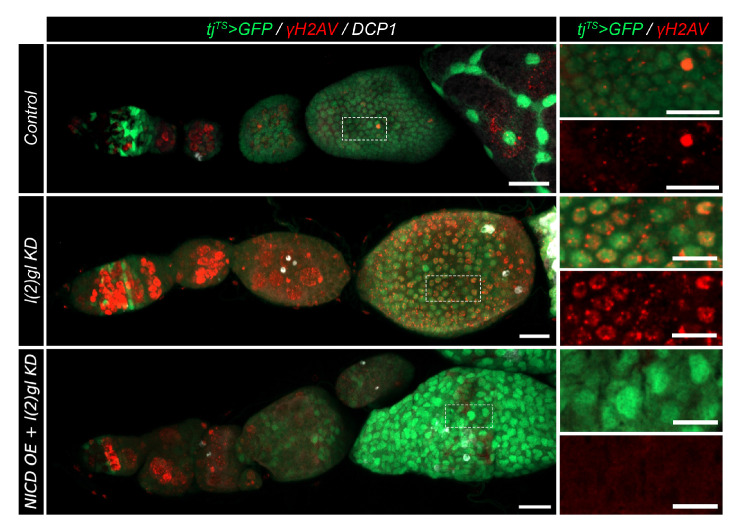
NICD overexpression protects from marks of DNA damage. Confocal images showing DNA damage marked with γ-H2AV antibody (red) in post-mitotic follicle cells (white box and ROI inset). Cell death is marked using DCP1 antibody staining (white). Scale bar = 10 μm.

**Figure A5:**
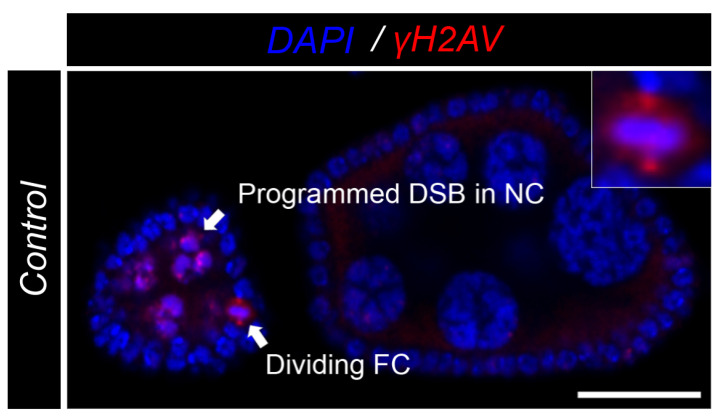
Control γ-H2AV signal in germline and dividing cells. Control egg chamber highlighting the expected double stranded break (DSB) of marked by γ-H2AV antibody in red (DNA) in the nurse cells in early egg chambers. Additional signal is observed in spindles and centrosomes of dividing cells. A subset of a dividing cell is present in the upper right corner. DAPI marks nuclei in blue. Scale bar = 20 μm.

Apoptosis serves as a protective measure for a tissue to eliminate damaged or abnormal cells. Often DNA damage induces cell death so we wanted to identify if NICD, l(2)gl +/− tumor cells were better able to survive because of protection from DNA damage. In *Drosophila*, DNA damage (specifically double-stranded breaks-DSBs) can be observed using the “gold standard” of detection, γ-H2AV antibody [32]. If the DNA has been damaged, this histone variant (H2AV) will be phosphorylated by protein kinases, such as ATM, ATR, and DNA- PK at the site of the break [33,34,35]. DSBs are known to occur, and be later repaired, in the germline during normal development (Figure A5). We also found that this antibody marked mitotic follicle cells during early oogenesis (Figure A5). To compare levels of DNA damage and resulting cell death in NICD, l(2)gl +/− and l(2)gl +/− tumors, we used γ-H2AV and DCP1 antibodies, respectively. We compared γ-H2AV signal in endocycle-staged egg chambers. NICD, l(2)gl +/− tumor cells had dramatically reduced γ-H2AV marks compared to l(2)gl +/− tumors (Figure 4).

### 3.4. Bulk RNA Sequencing Reveals Pre- and Post-Tumor Notch-Induced Targets Involved in DNA Damage and Cell Survival

**Figure 5:**
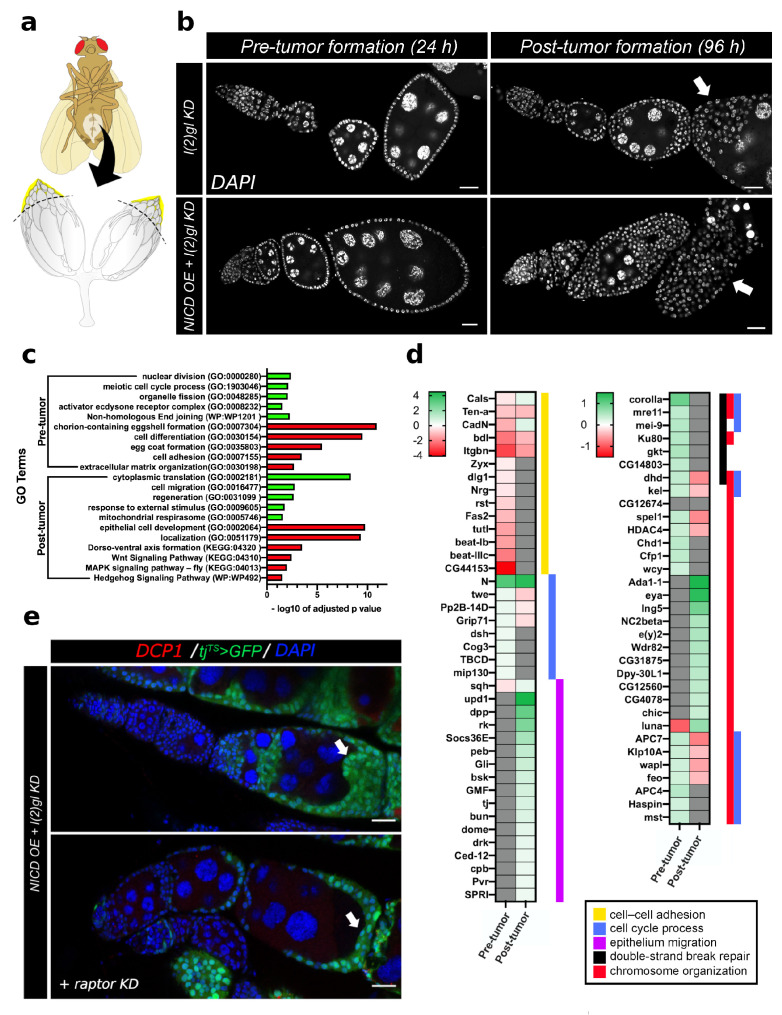
RNA sequencing reveals the Notch-induced genes regulating tumorigenesis and cell survival. (**a**) An illustration of sample collection method. Anterior ovaries, including egg chamber stages up to but not including stage 10A (highlighted in yellow) were collected for library preparation and sequencing. (**b**) Representative confocal images of tumor samples collected at either 24 h (after Gal4 induction but before tumor formation) and 96 h (after significant tumor formation). White (DAPI) marks nuclei. Arrows point to tumor overgrowth in both genetic backgrounds at 96 h. Scale bar = 20 μm. (**c**) Results from GO term enrichment analysis of pre- and post-tumor genes which were up or down regulated in NICD OE + l(2)gl KD samples compared to l(2)gl KD. Significantly enriched GO terms for upregulated (green) and downregulated (red) genes are shown plotted with respective negative log 10 of adjusted *p* values. (**d**) Heatmap highlighting expression patterns (LogFC) of select genes from pre- to post-tumor conditions. Colored bars to the right of each heatmap signify the GO term each gene belongs to: cell–cell adhesion (GO:0098609), cell cycle process (GO:0022402), epithelium migration (GO:0090132), double-strand break repair (GO:0006302), or chromosome organization (GO:0051276). (**e**) One gene identified, *raptor*, is upregulated in NICD OE tumor cells prior to tumor formation (0.57 LogFC) but downregulated following tumor formation (−0.51 LogFC). Consistent with a role in Notch-induced tumor formation, *raptor* knock-down (KD) in an NICD OE + l(2)gl KD background reduces tumor size (arrows). Cell death marked with DCP1 (red), DNA marked with blue), follicle cells expressing GFP (green (DAPI) to mark Gal4 expression. Scale bar = 20 μm.

Since Notch is a transcription factor, it is likely that nuclear NICD accumulation impacts countless downstream targets. To begin understanding the mechanism of Notch-induced cell survival and DNA damage response, we used bulk RNA sequencing in pre- and post-tumor tissue to determine which genes were regulated following NICD accumulation but before tumor formation and which genes were regulated following tumor formation. Pre-tumor tissues were collected from the anterior of dissected ovaries following 24 h of Gal4 activity and post-tumor tissues were collected following 96 h of Gal4 activity (Figure 5a,b). We then performed an Gene Ontology (GO) analysis to identify genes which were enriched for each dataset. Through this analysis of GO terms, we have identified that before tumors form, Notch promotes signals related to DNA damage repair and proliferation such as nuclear division, meiotic cell cycle process, and Non-homologous End joining (Figure 5c). Following tumor formation there was a secondary signaling response involving genes regulating translation, regeneration, response to external stimulus, and mitochondrial respirasome. As we would expect in tumor samples where oogenesis is disrupted, most of the downregulated genes in both time-points involve cell differentiation signals such as Wnt, MAPK, and Hh signaling pathways as well as genes regulating egg coat formation and chorion-containing eggshell formation (Figure 5c). We have specifically highlighted some of the genes within these GO categories to compare their expression across pre- and post-tumor formation time-points (Figure 5d). One gene which is upregulated before tumor formation is a conserved cell growth and metabolism gene in the TOR complex called *raptor*. In human renal cell cancer, *raptor* upregulation mediated the resistance of cancer cells to mTOR kinase inhibitors, promoting treatment resistance [36]. To experimentally validate that *raptor* early in tumorigenesis is involved in Notch-induced tumor growth, we knocked down *raptor* with RNAi in the NICD, l(2)gl +/− tumors. We find that even by three days of growth, the tumor growth phenotype is partially rescued indicating that *raptor* is indeed involved in tumor formation in the follicle cells when Notch is overexpressed (Figure 5e).

### 3.5. Single-Cell RNA Sequencing Analysis Identifies Cell-Type-Specific Responses to Ectopic Nicd

**Figure 6:**
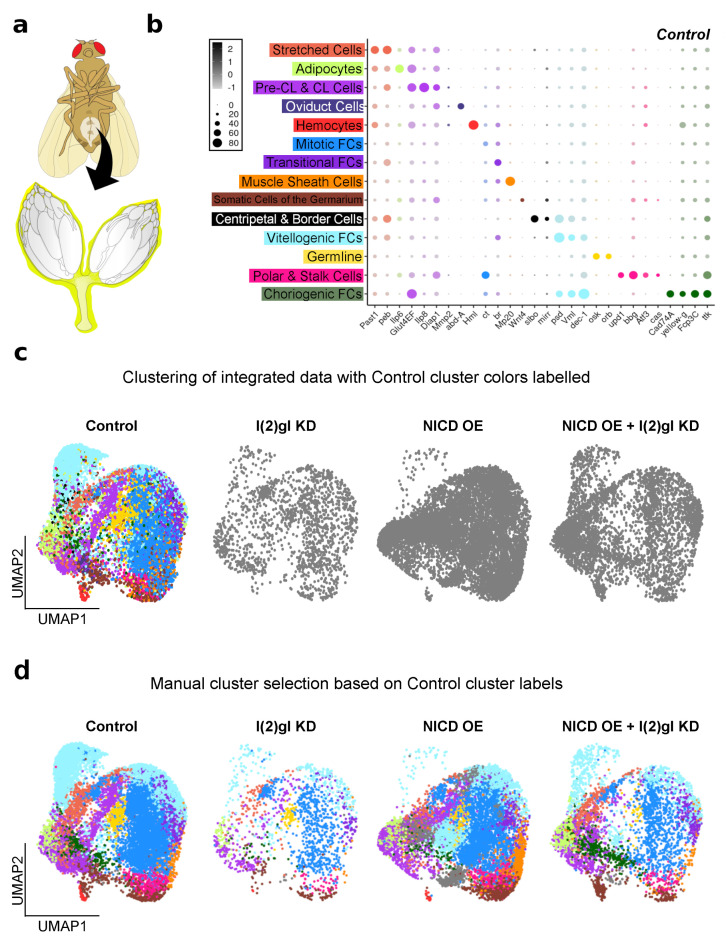
Single-cell RNA sequencing of tumorigenic follicle cells. (**a**) An illustration of sample collection. Entire ovaries and interconnected oviduct tissue (highlighted in yellow) was sampled for sequencing. (**b**) Dot plot showing the known marker genes used to identify the cell types in each control cluster. Average expression (from −1 to 2 LogFC) and percentage of cells expressing each marker (from 0 to 80%) is shown. (**c**) UMAP plot showing single cells (dots) clustered together by shared RNA expression profile. Clusters identified (and colored) in b are shown in the control dataset which was integrated with the other 3 datasets and clustered together (shown in gray). (**d**) Clusters were manually selected based on the cell identities shown in c and colored to compare cluster type between all genotypes. As expected clusters with late-stage follicle cell sub-types such as Vitellogenic and Choriogenic follicle cells (FCs) are reduced in the l(2)gl KD sample. The NICD OE sample has three unique clusters that could not be identified and remain in gray.

During oogenesis, the follicle cells differentiate into different sub-types that have differing transcriptional profiles as demonstrated by previous single-cell RNA sequencing studies [15,37,38]. This heterogeneity in expression between cell types is not detectable in bulk RNA sequencing experiments. To determine how NICD OE impacts signaling in just the follicle cell subgroups of interest within NICD, l(2)gl +/− tumors, we performed single-cell RNA sequencing on whole ovaries from control, NICD OE, l(2)gl KD, and NICD OE + l(2)gl KD samples after Gal4 induction for 3 days in permissive temperature. Other interconnecting tissues were also sampled including: hemocytes, muscle sheath cells, adipocytes, and oviduct (Figure 6a,b). This was to preserve the ovary sample of interest as these connecting tissues are technically challenging to separate mechanically. Instead, signals from these cells were identified and separated during downstream analysis using genetic markers. Previously identified biological markers were used to determine the identity of each cell type in the Uniform Manifold Approximation and Projection (UMAP) plot [15] (Figure 6b,c). Integrated markers across all samples were used to ensure that similar cell types between genotypes were clustered together and the annotated control dataset was used to identify previously reported cell-type markers across the four different genotypes (Figure 6b–d).

**Figure 7:**
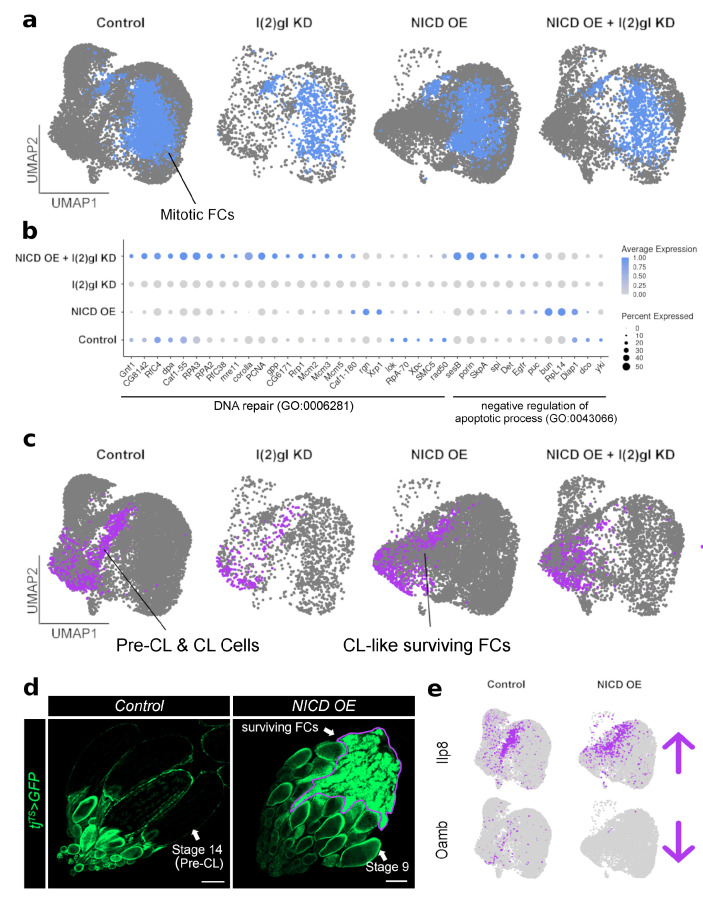
Single-cell RNA sequencing reveals cell-type-specific expression patterns of survival-related genes following NICD overexpression. (**a**) UMAP plot of all four genotypes with the mitotic follicle cells (FCs) specifically highlighted in blue. These cells were subset for analysis in (**b**). (**b**) Dot plot of mitotic FC cluster split between each genotype. DNA repair (GO:0006281) and negative regulation of apoptotic process (GO:0043066) genes are grouped by GO term. NICD OE + l(2)gl KD cells have a higher expression of these survival-related genes compared to l(2)gl KD. Some genes, like *rgn*, *Xrp1*, *bun*, *RpL14*, and *Diap1* are more highly expressed in NICD OE cells. (**c**) To more carefully examine surviving cells in NICD OE genotype, the Pre-CL and CL Cells cluster is highlighted in purple and subset for analysis in (**e**). (**d**) Confocal images of control and NICD OE ovaries. Unlike control oogenesis, NICD OE blocks development at stage 9, where the germline undergoes apoptosis and the follicle cells survive in the posterior ovary. Scale bar = 100 μm. (**e**) Feature plots showing that like corpus luteum (CL) cells in the control sample, the surviving NICD OE follicle cells have upregulated the insulin pathway gene, *Ilp8*, expression. However, unlike true CL cells, CL-like surviving cells lack expression of the ovulation gene, *Oamb*.

With single-cell RNA-sequencing we were able to subset the mitotic FCs which form the tumor overgrowths in vivo (Figure 7a). Without conflicting signals from expression of other cell types, we were able to identify the most highly expressed genes in the cluster grouped into DNA repair genes and negative regulation of apoptotic process genes. These genes are most highly expressed in the NICD and NICD, l(2)gl +/− genotypes (Figure 7b).

**Figure A6:**
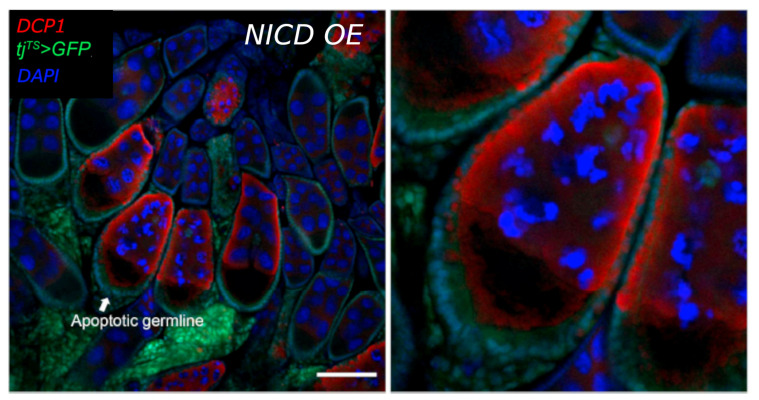
Germline apoptosis occurs following NICD overexpression in follicle cells. Confocal image (left) of an ovary from the NICD OE condition. Follicle cells are marked by expression of GFP driven by tj-Gal4 (green). DCP1 antibody (red) marks apoptosis in the germline (nurse cells and oocyte). DAPI marks nuclei (blue). A single mid-staged egg chamber at the mid-oogenesis checkpoint is shown with apoptotic germline and shown in a close up image at right. Scale bar = 20 μm.

Using the single-cell dataset we were also able to identify that NICD overexpression alone promotes a different kind of cell survival than in an l(2)gl +/− tumor background. We identified that the Pre-corpus luteum (CL) and CL cell cluster is robust in the NICD OE sample (Figure 7c). Since the corpus luteum cells naturally persist in the egg chamber following ovulation, we wondered if NICD OE in follicle cells was promoting a CL-like state promoting cell survivial and if this could explain how NICD OE allograt tissue survived as well as NICD, l(2)gl +/− tissue. To examine this more closely, we imaged entire ovaries expressing NICD OE without breaking apart the ovarioles to look for CL-like cells (Figure 7d). This revealed that by stage 9, the germline has completely undergone apoptosis (Figure A6) and the follicle cells, which express NICD, accumulate in the posterior of the ovary similar to corpus luteum structures left behind after ovulation(Figure 7d). Single-cell expression data shows that these "CL-like” surviving cells had increased *Ilp8* expression, an insulin pathway gene involved in relaying growth status. However in contrast, unlike control CL cells, the gene, *Oamb*, which is essential for ovulation, is downregulated (Figure 7e).

## 4. Discussion

In this study, we established a system for the investigation of ectopic NICD accumulation in tumor cell nuclei, in vivo, using adult *Drosophila* follicular epithelium in an l(2)gl KD background. Our findings recapitulate the nuclear accumulation and cell survival phenotypes observed in human cancer types, making this system a valuable tool for interrogating the effects of NICD overexpression during tumorigenesis [6,39,40].

Often diagnoses of disease progression are made based on observations of morphological abnormalities in order to determine if cells are benign or malignant [41]. These abnormalities often include larger nuclear size. We observed that NICD accumulation impacted the morphology and size of tumor cell nuclei without impacting ploidy. These results are consistent with the current understanding that nuclear size is often one of the key identifying features of malignant cells and that in many types of human carcinoma such as those originating from the bladder, colon, breast, lung, skin, cervix, and prostate, nuclear size is not well correlated with ploidy [41,42,43].

We also found that NICD, l(2)gl +/− tumor cells had dramatically reduced DNA damage compared to tumor controls and bulk RNA sequencing data confirmed that many DNA-binding and damage response genes are altered following NICD accumulation. This is an interesting result given the observation that NICD OE increases the size of nuclei. Previous work has shown that decondensation of chromatin promotes the repair of various types of DNA damage including double-stranded breaks [44]. It is possible that this is the mechanism by which DNA damage is repaired in tumors with nuclear NICD accumulation, promoting survival and proliferation.

RNA sequencing demonstrates the complexity of downstream transcriptional changes from NICD accumulation in tumor cell nuclei. Despite this, clear patterns emerge demonstrating that Notch influences core pathways involved in regulating DNA damage repair, genome organization and cell cycle. Additionally, patterns of gene expression like *raptor*, where upregulation occurs before tumor formation, followed by downregulation suggest that there are primary and secondary impacts of Notch expression in a tumor background. There appear to be more specialized genes that are upregulated following tumor formation that point to an epithelial to mesenchymal transition in the follicle cells. For example, we have identified many genes typically involved in epithelial migration, response to stimulus, and localization genes which are upregulated only following tumor formation. These will provide many potential targets for future research into the impact of NICD on tumorigeneis and tumor dynamics.

## Figures and Tables

**Figure A4:**
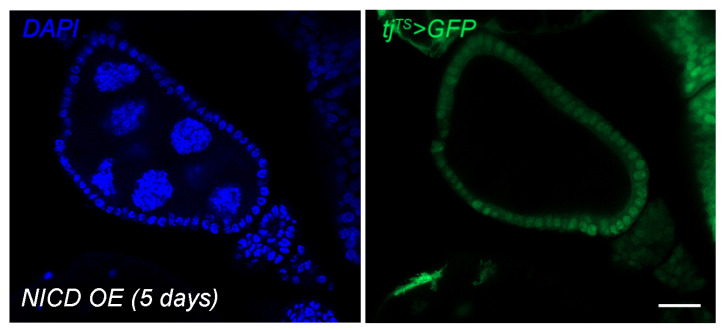
NICD overexpression in the follicle cells after five days in 29 ${}^{\circ }$C. Mid-stage egg chamber from NICD OE genotype following five days in 29 ${}^{\circ }$C for allograft experiment. Similar to three days in 29 ${}^{\circ }$C, there is no tumor formation. Scale bar = 10 μm.

## Data Availability

All sequencing data (raw and processed) can be found in the Gene Expression Omnibus (GEO) database (GSE182505).
